# Neutrophil extracellular traps promote lipopolysaccharide-induced airway inflammation and mucus hypersecretion in mice

**DOI:** 10.18632/oncotarget.24022

**Published:** 2018-01-08

**Authors:** Yong Zou, Xi Chen, Jian Xiao, Dong Bo Zhou, Xiao Xiao Lu, Wei Li, Bin Xie, Xiao Kuang, Qiong Chen

**Affiliations:** ^1^ Department of Emergency Medicine, Xiangya Hospital of Central South University, Changsha, China; ^2^ Department of Respiratory Medicine, Xiangya Hospital of Central South University, Changsha, China; ^3^ Department of Geriatrics, Respiratory Medicine, Xiangya Hospital of Central South University, Changsha, China

**Keywords:** neutrophil extracellular traps, lipopolysaccharide, airway inflammation, mucus hypersecretion, TLR4/NF-κB

## Abstract

Bacterial lipopolysaccharide (LPS) contributes to airway inflammation and mucus hypersecretion in chronic airway inflammatory diseases, such as chronic obstructive pulmonary disease (COPD) and cystic fibrosis (CF). Neutrophil extracellular traps (NETs) are extracellular meshworks composed of DNA fibers and antimicrobial proteins. Although NET formation has been detected in COPD and CF patients, how NETs contribute to these diseases is poorly understood. This study was performed to clarify the effects and mechanisms of action of NETs in airway inflammation and mucus hypersecretion. We created a murine model of LPS-induced airway inflammation and mucus hypersecretion, and found that LPS-induced NET formation was degraded by aerosolized DNase I treatment in mice. Degradation of NETs by aerosolized DNase I reduced LPS-induced airway inflammation and mucus hypersecretion in mice, this reduction correlated with suppression of TLR4/NF-κB signaling pathway. More importantly, NETs promoted LPS-induced production of IL-1β, IL-6 and TNF-α in macrophages. These results suggest NET degradation using aerosolized DNase I is a potential new therapeutic strategy for treating COPD and CF.

## INTRODUCTION

Neutrophil extracellular traps (NETs) are extracellular meshworks composed of decondensed chromatin and characteristic granule proteins, such as histones, myeloperoxidase (MPO), neutrophil elastase (NE) [1]. NETosis or NET formation is a distinct and complicated cell death process that differs from apoptosis and necrosis [1, 2]. First, neutrophils are activated by infective factors or ‘sterile’ stimuli, such as autoantibodies, cholesterol crystals or cytokines. Then, citrullination of histone3 (cit-H3) and heterochromatin decondensation cause NETs formation, and peptidyl arginine deiminase 4 (PAD4) catalyzes this process [3, 4]. Finally, with the help of intracellular calcium and MPO, decondensed chromatin and cytosolic protein particles are mixed and NETs formed intracellularly are released to outside of the cell [5]. NETs are ultimately cleared by DNase I degradation and macrophages phagocytosis [6, 7].

NETs were initially described as antimicrobial molecules that could trap and kill pathogens, preventing the dissemination of infections [1]. However, when excessive NETs are formed or can not be cleared timely, harmful effects can occur. For example, NET-bound components contribute to the development of many systemic autoimmune diseases, such as rheumatoid arthritis, systemic lupus erythematosus and small vessel vasculitis [8–11]. Recent studies have also found that NETs have pro-inflammatory effects. Warnatsch et al. [12] found increased levels of NETs in a murine model of atherosclerosis. NETs drove atherosclerosis by increasing cytokine production; this pro-inflammatory effect was markedly suppressed by DNase I treatment and by knocking out the NE/PR3 gene (two important components of NETs). Moreover, NETs primed macrophages for cytokine release *in vitro* [7, 12, 13].

Lipopolysaccharide (LPS), which is a critical component of the outer membrane of gram-negative bacteria, is a major factor in the development of Chronic Obstructive Pulmonary Disease (COPD) [14, 15] and cystic fibrosis pulmonary disease (CF) [16, 17]. Airway inflammation and mucus hypersecretion are the main pathologic features of COPD [18, 19] and CF [20, 21]. In addition, persistent NET formation has been found in patients with COPD [22–24] and CF [25, 26]. However, how NETs affect airway inflammation and mucus hypersecretion in COPD or CF is unclear.

Intratracheal administration of LPS in mice is a widely used animal model to study airway inflammation and mucus hypersecretion [27–29]. The sudy was undertaken to explore the effects of NETs on airway inflammation and mucus hypersecretion in an LPS-induced acute model, and to determine the possible molecular mechanisms that underlie these effects.

## RESULTS

### LPS-induced NET formation was degraded by aerosolized DNase I in mice

**Figure 1:**
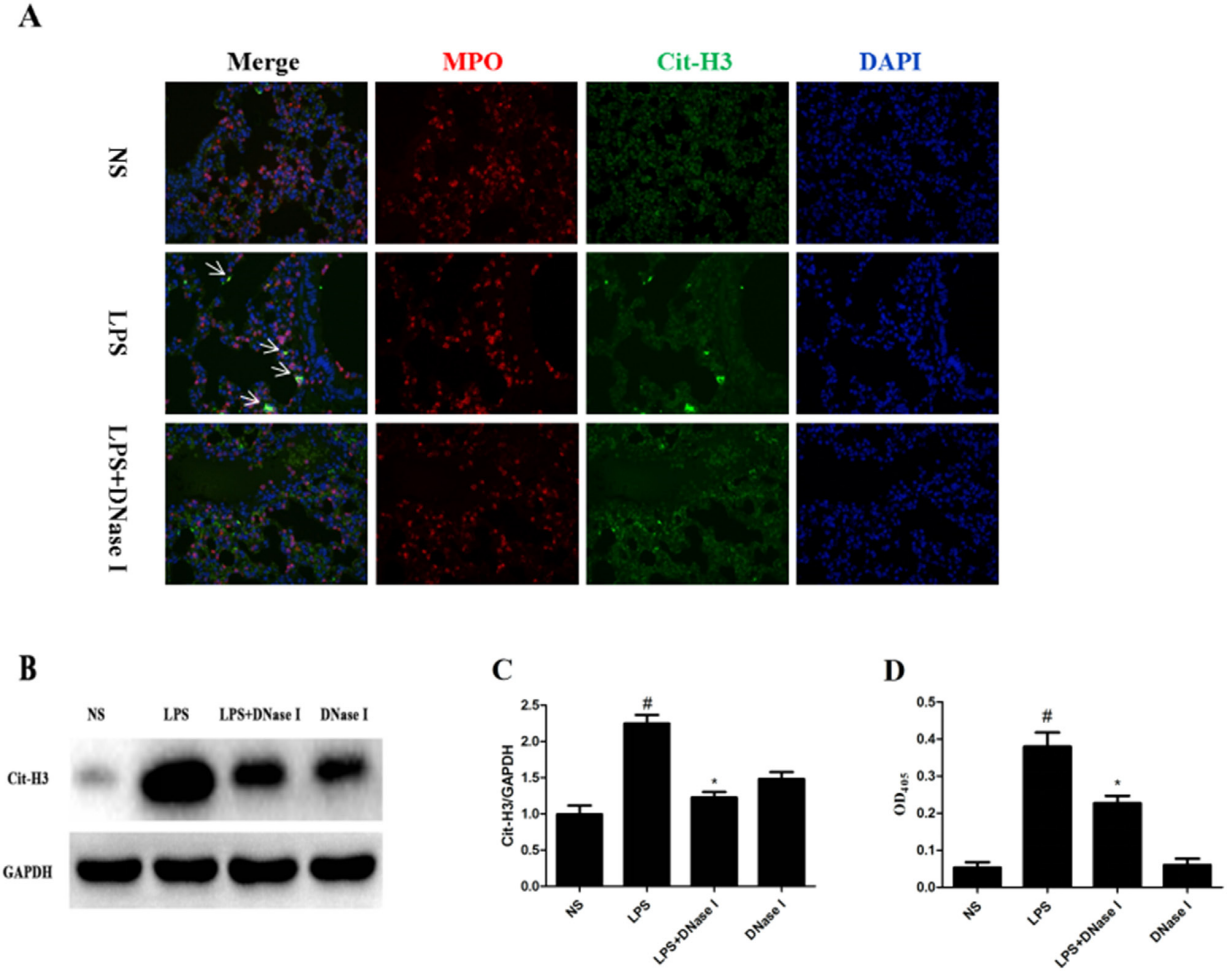
LPS-induced NET formation was degraded by aerosolized DNase I in mice Mice received aerosolized DNase I (120 U of DNase I in 5mL of normal saline [NS]) or NS at 4 and 12 hours after LPS injection (2 mg of LPS in 50μL NS). **(A)** Representative images (×200) show merged neutrophil DNA (blue), Cit-H3 (green), and MPO (red) via confocal microscopy of lung sections from mice in the NS, LPS and LPS plus DNase I groups. Arrows show NET formation. **(B, C)** Representative Western blots and quantification of Cit-H3 protein expression in lung homogenates of all groups. **(D)** BALF levels of NET-DNA were detected using an MPO-DNA ELISA kit. ^*^P <.05 compared with NS group. ^#^*P* <.05 compared with LPS group. Data represent the mean ± SD (n=6).

To determine whether NET formation was induced by LPS in mice, we first analyzed the composition of NETs using immunofluorescence staining of lung tissues. LPS, but not normal saline (NS), induced NET formation based on the co-localization of major structural components of NETs, including DNA, MPO and cit-H3 (Figure 1A). However, we did not observed the typical fiber mesh structure of NETs in the LPS-treated group, which may be due to destruction of lung tissue structure, inappropriate tissue section or the presence of short fragments of DNA-protein complexes in the airways [30]. Thus, Western blot was performed to determine the lung tissue cit-H3 levels, a key marker indicating NET formation. Cit-H3 levels were significantly higher in the LPS group than NS group (Figure 1B, 1C). Moreover, NET formation was significantly greater in the LPS group than NS group, which was confirmed by measuring the levels of MPO-DNA complexes in broncho alveolar lavage fluid (BALF) using a capture enzyme-linked immunosorbent assay (ELISA) kit (Figure 1D). However, LPS-induced NET formation was degraded by aerosolized DNase I (Figure 1).

### Aerosolized DNase I decreased LPS-induced airway inflammation in mice

**Figure 2:**
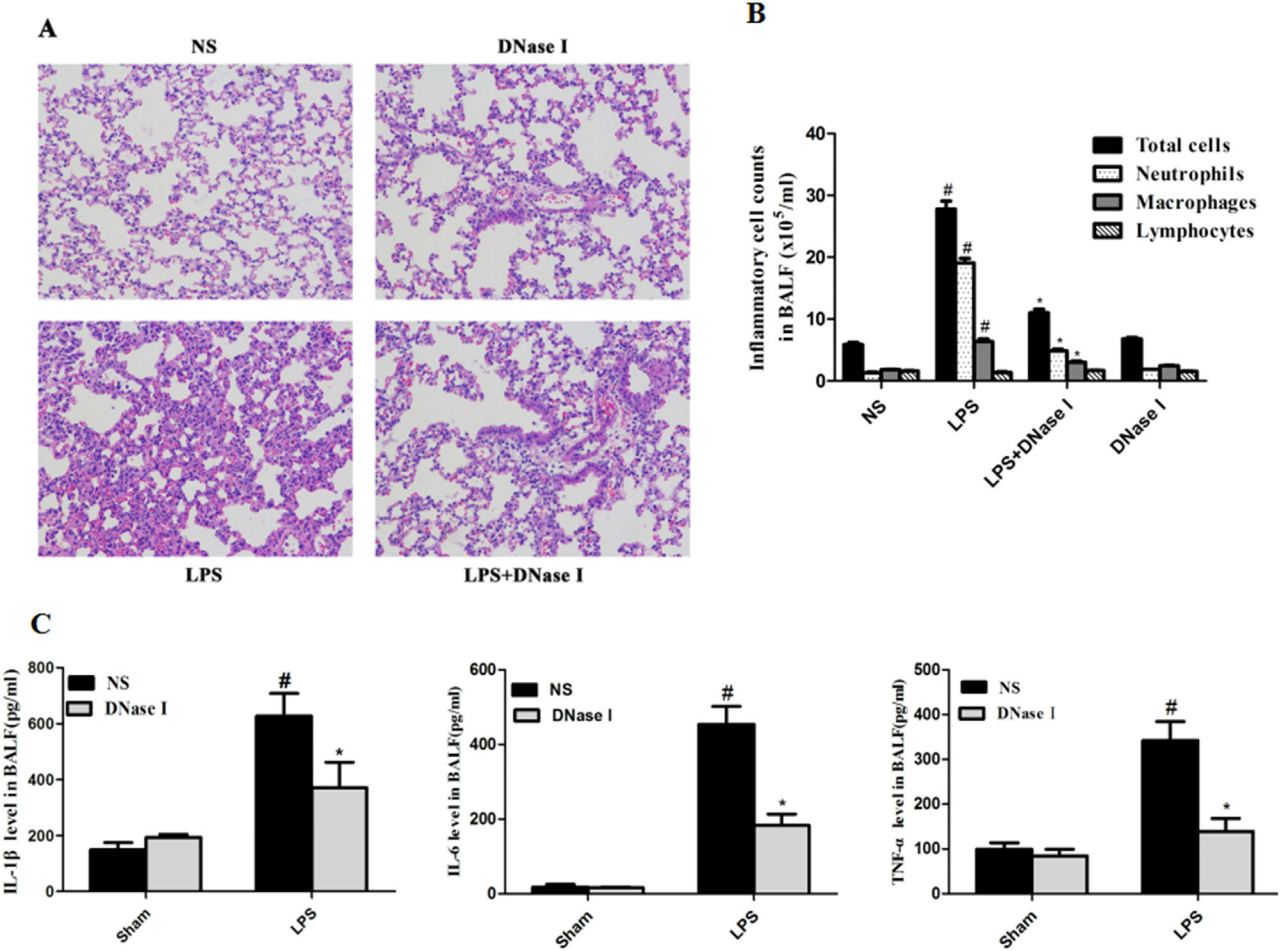
Degradation of NETs by aerosolized DNase I decreased LPS-induced airway inflammation in mice Mice received aerosolized DNase I (120 U) or NS at 4 and 12 hours after LPS injection. The lung tissues and BALF from the mice were collected 24 hours after LPS administration. **(A)** Representative figures (×200) ofhematoxylin and eosin staining in mouse lung tissues. **(B)** Total cells, neutrophils, macrophages and lymphocytes in BALF were counted under × 400 magnification using a light microscope. **(C)** MouseTNF-α, IL-6 and IL-1β levels in BALF were measured using ELISA. Data represent the mean ± SD (n=6). ^*^P <.05 compared with control. ^#^*P* <.05 compared with LPS.

Next, we investigated the effects of NET formation on LPS-induced airway inflammation. Previous studies have shown that DNase I degrades NET formation *in vivo* and *in vitro* [12, 31]. This was consistent with our results, which showed that aerosolized DNase I degraded NET formation in our LPS-induced mouse model (Figure 1). Hematoxylin and eosin (H&E) staining of lung tissue from mice found that aerosolized DNase I suppressed LPS-induced lung inflammation (Figure 2A). LPS-treated mice had significantly higher levels of inflammatory cells in the BALF, especially neutrophil than the NS-treated mice, and this effect was almost abolished by aerosolized DNase I (Figure 2B). Subsequently, we explored the effects of NETs on the production of proinflammatory cytokines in LPS-induced mice. LPS-induced increases in IL-1β, IL-6 and TNF-α in the BALF were dereased by aerosolized DNase I (Figure 2C).

### Aerosolized DNase I decreased LPS-induced mucus hypersecretion in mice

**Figure 3:**
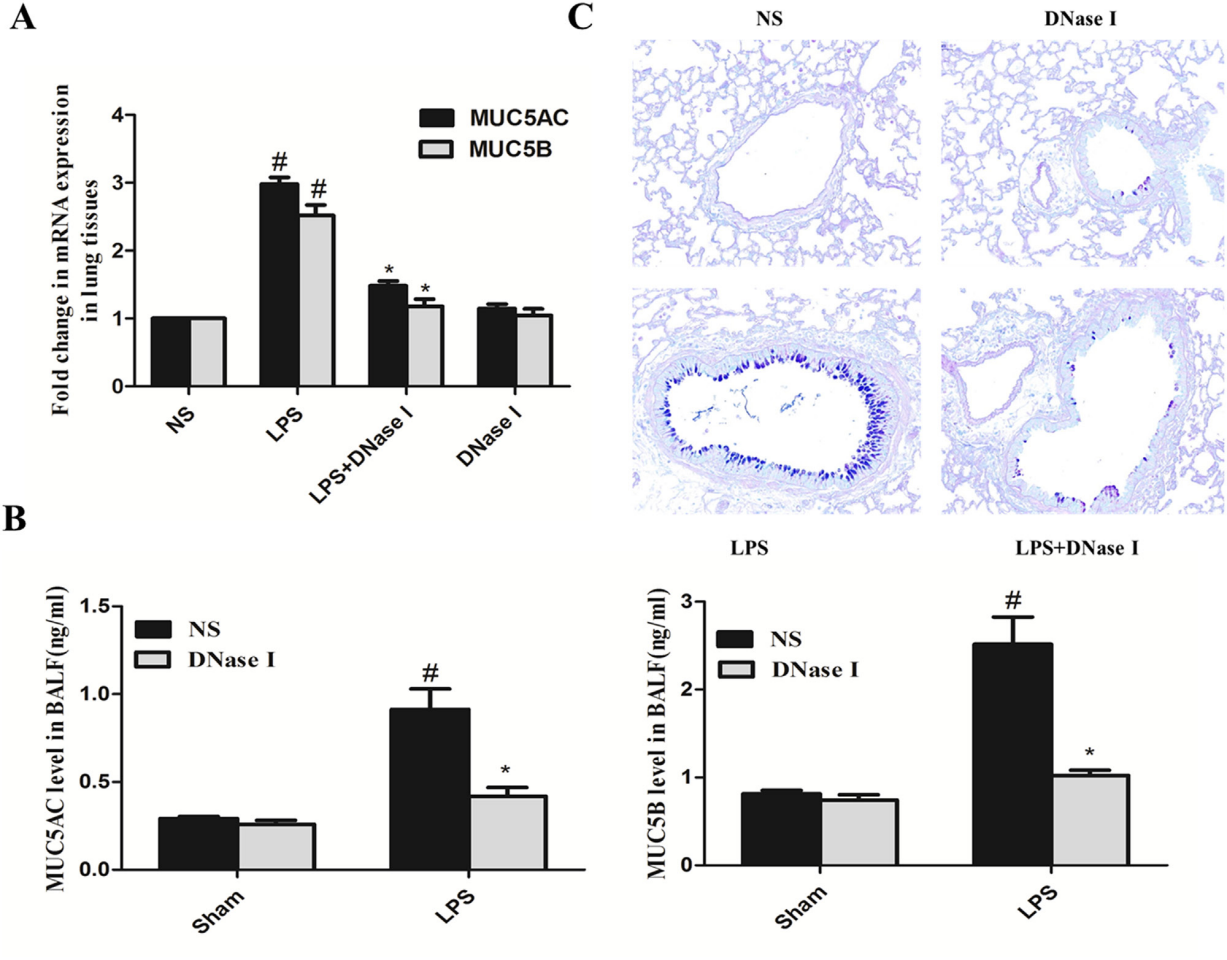
Degradation of NETs by aerosolized DNase I decreased LPS-induced mucus hypersecretion in mice Mice received aerosolized DNase I (120 U) or NS at 4 and 12 hours after LPS injection. The lung tissues and BALF from the mice were collected 24 hours after LPS administration. **(A)** MUC5AC and MUC5B mRNA levels in lung tissue from each group were detected by quantitative-RT-PCR. **(B)** MUC5AC and MUC5B secretion in BALF was measured by ELISA. **(C)** Representative figures (×200) of AB-PAS staining in mouse lung tissues. Data represent the mean ± SD (n=6). ^*^P <.05 compared with control. ^#^*P* <.05 compared with LPS.

We also explored the effects of NET formation on LPS-induced mucus hypersecretion in mice. LPS increased MUC5AC and MUC5B mRNA expression in mouse lung tissue and this effect was abrogated by aerosolized DNase I (Figure 3A). In addition, aerosolized DNase I decreased LPS-induced MUC5AC and MUC5B secretion (Figure 3B), two critical gel-forming mucins in the airway [32, 33]. Alcian Blue-Periodic Acid Schiff (AB-PAS) staining of mouse lung tissue also demonstrated that aerosolized DNase I decreased LPS-induced goblet cell metaplasia (Figure 3C).

### Aerosolized DNase I suppressed activation of the TLR4/NF-κB signaling pathway after LPS exposure in mice

**Figure 4:**
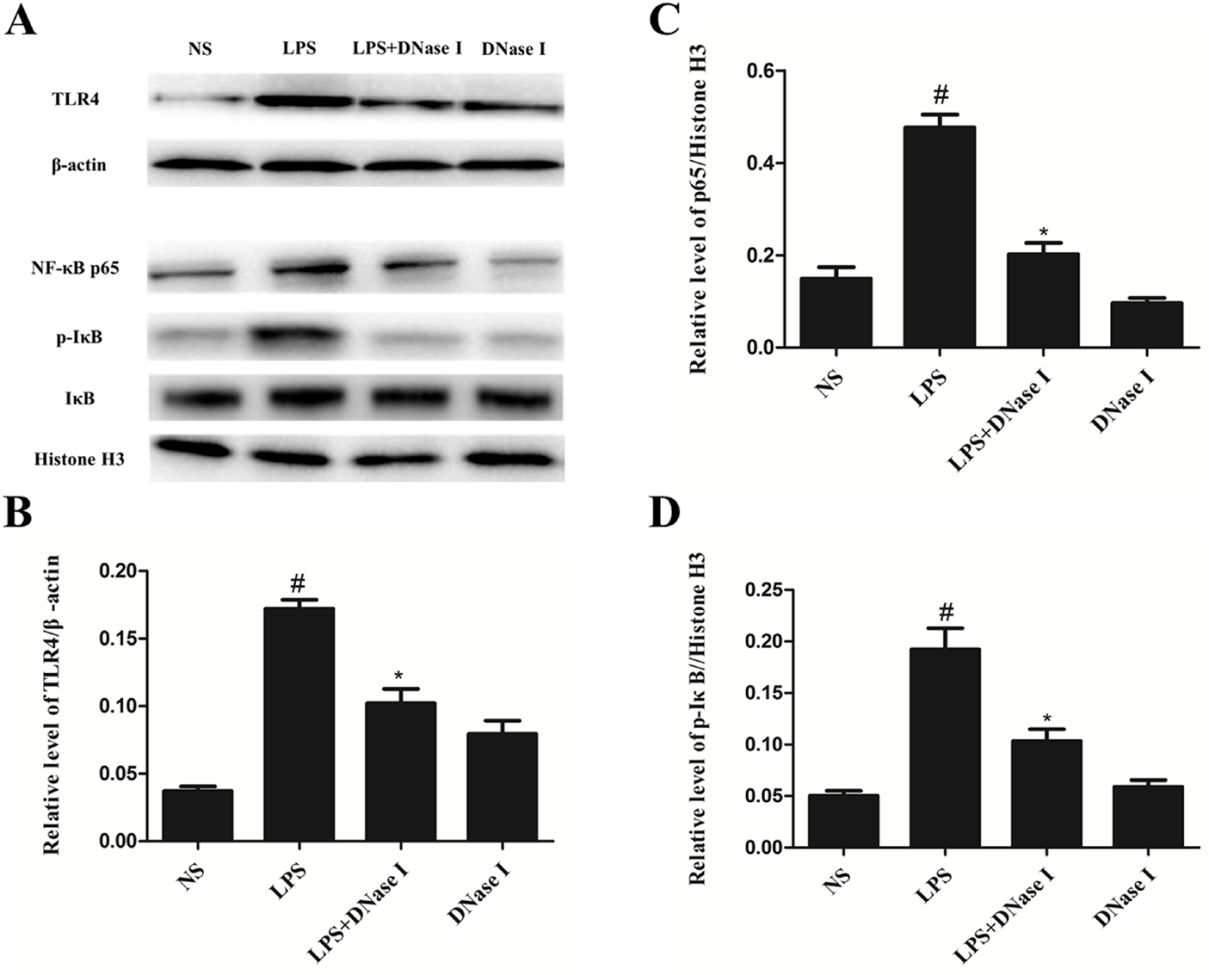
Degradation of NETs by aerosolized DNase I suppressed activation of the TLR4/NF-κB signaling pathway after LPS exposure in mice Mice received aerosolized DNase I (120 U) or NS at 4 and 12 hours after LPS injection. Lung tissues from the mice were collected 24 hours after LPS administration. **(A)** Representative Western blots of TLR4, β-actin, NF-κB p65, p-IKB-α, IKB-α, and histone H3 in lung tissue from each group. **(B, C, D)** Quantification of Western blot data from A. Data represent the mean ± SD (n=6). ^*^P <.05 compared with control. ^#^*P* <.05 compared with LPS.

We explored possible mechanisms of action of NET formation on LPS-induced airway inflammation and mucus hypersecretion in mice. Because previous studies have shown that TLR4/NF-κB signaling pathway is involved in inflammation and mucus hypersecretion [34, 35] and *in vivo* and *in vitro* studies have demonstrated the proinflammatory effects of NETs [7, 12, 13]. We speculated that NETs affect LPS-induced airway inflammation and mucus hypersecretion in mice via the TLR4/NF-κB signaling pathway. Western blot analyses demonstrated that TLR4 levels in lung tissue were much higher in the LPS group than the NS group, however, aerosolized DNase I significantly suppressed LPS-induced TLR4 expression (Figure 4A), demonstrated by the ratio of TLR4 to β-actin (Figure 4B). Similarly, p-IκB-α and NF-κB p65 protein expression was significantly greater in the LPS group than the NS group, and again, aerosolized DNase I inhibited this LPS-induced increased expression (Figure 4A, 4C, 4D). Thus, these results indicated that NETs increased LPS-induced TLR4, p-IκB-α and NF-κB p65 expression in mouse lung tissues.

### NETs promote LPS-induced cytokine production in macrophages

**Figure 5:**
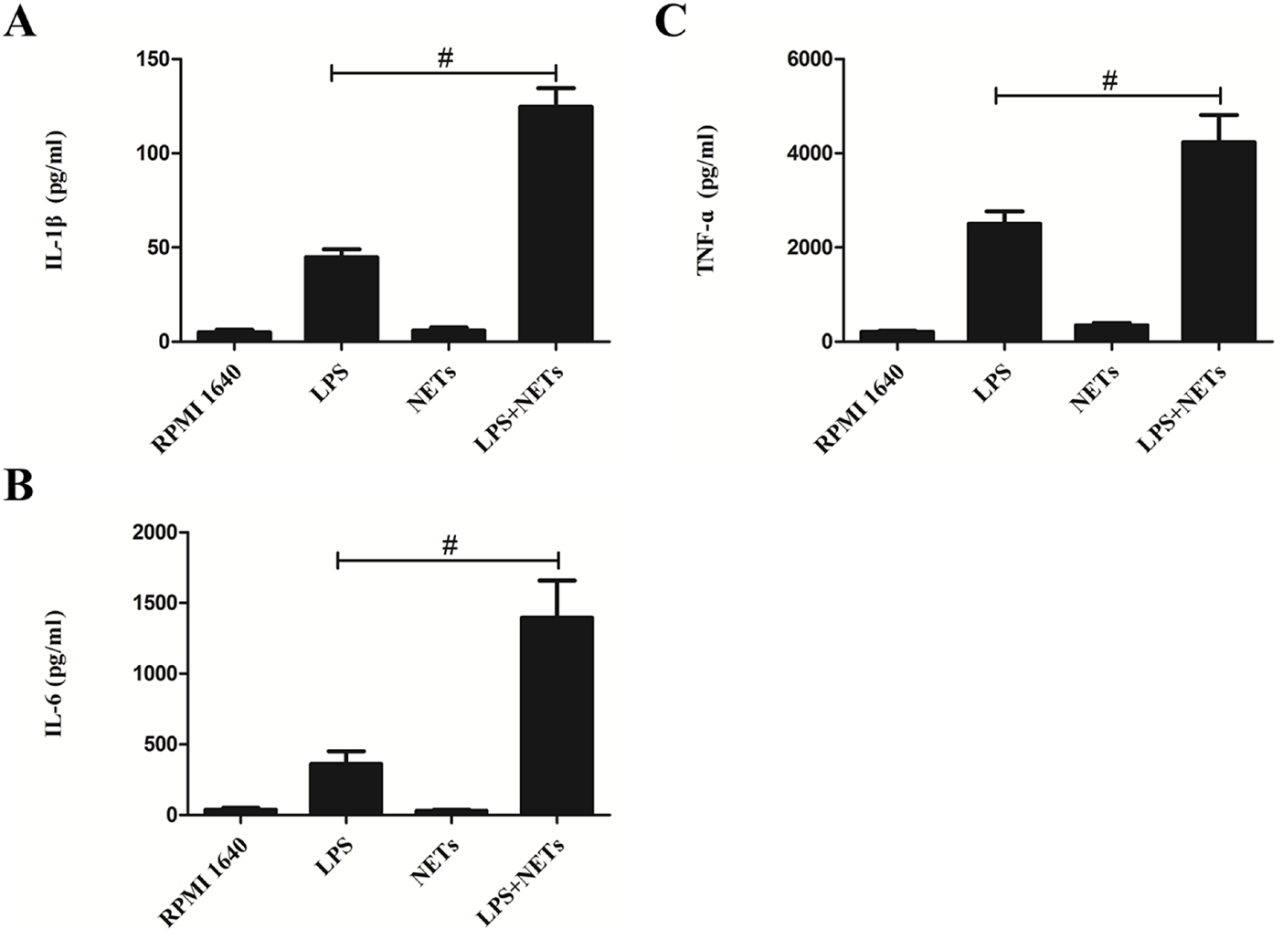
NETs promote LPS-induced cytokine production in macrophages Neutrophils from healthy donor whole blood were isolated and purified by density gradient separation technique. Purified neutrophils were stimulated by 500 nM of PMA and cell-free NETs were obtained and quantified. CD14 blood-derived human monocytes were obtained by magnetic cell sorting and differentiated into macrophages (HMDMs) by adding 100 ng/mL of recombinant human M-CSF. HMDMs were treated with LPS (100 ng/mL) or NETs (20 ng/μL) alone or together. Supernatants were recovered after 24 hours and used to detect IL-1β **(A)**, IL-6 **(B)** and TNF-α **(C)** levels by ELISA kits. Data represent the mean ± SD (n=3). ^#^*P* <.05 compared with LPS.

To explore how NETs affect LPS-induced airway inflammation, we prepared NETs from PMA-stimulated human neutrophils and investigated their effect on macrophages *in vitro*. Stimulation with NETs alone yielded minor increases in IL-1β and TNF-α concentrations in culture supernatants (*P* >.05; Figure 5). However, concentrations of IL-1β, IL-6 and TNF-α were significantly greater in macrophage culture supernatants stimulated with both NETs and LPS than in culture supernatants stimulated with LPS alone (*P* <.01; Figure 5).

## DISCUSSION

This study has several important findings. First, intratracheal injection of LPS induced NET formation that was degraded effectively by aerosolized DNase I. Second, aerosolized DNase I decreased LPS-induced airway inflammation and mucus hypersecretion in mice. Third, NETs promoted LPS-induced secretion of inflammatory cytokines in macrophages. Finally, NETs increased airway inflammation and mucus hypersecretion in mice and this was involved in the TLR4/NF-κB signaling pathway.

NETs are web-like networks of expelled DNA decorated with histones and various antibacterial proteins, such as MPO and NE [1]. Previous studies have shown that NET formation is induced by neutrophil exposure to microorganisms, including bacteria [36], viruses [37], fungi [38], parasites [39] and their products. Other studies have demonstrated that LPS, a prominent component of the outer membrane of gram-negative bacteria, induces NET formation in mice [31, 40], which is consistent with our results. To degrade the NETs induced by LPS in mice, we chose aerosol administration of DNase I rather than intratracheal instillations of DNase I based on the fact that intratracheal instillations of DNase I twice a day can cause mouse asphyxia. Moreover, in a placebo-controlled clinical trial of patients with CF, aerosolized recombinant human DNase I administered twice daily was shown to reduce the risk of infection, and improve pulmonary function and patient well-being [41]. Thus, aerosolized DNase I may be a more feasible and effective way to degrad the NETs.

Although NETs released by activated neutrophils can ensnare and kill microbes, and prevent the dissemination of infections, excessive formation of NETs often causes tissue damage and organ dysfunction [6, 42]. Recently, NET-mediated pro-inflammatory effects have been reported in *in vivo* and *in vitro* experiments [7, 12, 13]. In addition, increased NET formation was detected in COPD [22–24] and CF patients [25, 26]. Therefore, we postulated that airway inflammation and mucus hypersecretion, two common pathophysiologic features in patients with COPD or CF [18–21], may be increased by NETs. This hypothesis was confirmed in this study, which demonstrated that NET degradation by aerosolized DNase I suppressed LPS-induced airway inflammation and mucus hypersecretion in mice. In this study, LPS injection resulted in neutrophil recruitment and inflammatory cytokine secretion into the airways of mice, and these effect were suppressed by aerosolized DNase I, indicating the pro-inflammatory role of NETs in this process. To further clarify the target cell of NETs action, we stimulated macrophages by NETs, LPS, or NETs plus LPS and found that NETs significantly increased LPS-induced cytokine secretion. In the absence of LPS, NETs did not directly induce the secretion of inflammatory cytokines in macrophages. A recent article indicated that NETs promoted transcription of immature IL-1β in macrophages [12]. Thus, we theorized that NETs could induce transcription of inflammatory cytokines, but the maturation and release of inflammatory cytokines depended on other stimuli, such as LPS. This remains to be determined in future studies.

MUC5AC and MUC5B, two prominent gel-forming mucins, are secreted by airway epithelial cells stimulated by LPS [18]. Our data showed that LPS-induced MUC5AC and MUC5B mRNA and protein expression and goblet cell metaplasia were decreased by aerosolized DNase I in mice. Our study helped to identify the effect of NETs on airway mucus hypersecretion, which involves two possible mechanisms: 1) NETs act directly on airway epithelial cell receptors to induce MUC5AC and MUC5B secretion; 2) NETs prime macrophages for release of IL-1β and TNF-α, two cytokines that promote mucus hypersecretion [43, 44]. The mechanisms of NETs-mediated mucus hypersecretion are underway in our team.

TLR4, an important member of the TLR family of proteins known to regulate inflammation, recognizes LPS and triggers downstream signaling cascades [45]. Of note, the TLR4-mediated NF-κB signaling pathway plays key regulatory roles in inflammation and mucus hypersecretion [34, 35]. A recent study also found that degradation of NETs by DNase I treatment decreased IL-1β and IL-6 release, indicating that the pro-inflammatory effect of NETs is mediated by TLR4 [12]. Therefore, the mechanism by which NETs increase LPS-induced airway inflammation and mucus hypersecretion might be activation of the classic TLR4/NF-κB signaling pathway. We also found that degradation of NETs by aerosolized DNase I suppressed TLR4, p-IκB-α and NF-κB p65 protein expression in lung tissues.

In conclusion, this study demonstrated that LPS-induced NET formation was degraded by aerosolized DNase I in mice and that degradation of NETs by aerosolized DNase I decreased LPS-induced airway inflammation and mucus hypersecretion in mice. Macrophages may be the most important target cells for NET-induced pro-inflammatory effects. Because NETs promote airway inflammation and mucus hypersecretion, aerosolized DNase I might be a treatment option in patients with COPD or CF.

## MATERIALS AND METHODS

### Mice

The Animal Ethics Committee of Central South University (Changsha, China) approved this study. Specific pathogen-free C7BL/6 mice (6 to 8 weeks old, weighing 18 to 22g, male) were purchased from the Medical Experimental Animal Center of Central South University. Mice were kept under a 12-hour night–day rhythm with free access to standard rodent chow and water. All experimental procedures were performed in accordance with experimental animal guidelines.

### Animal exposure to LPS and DNase I administration

LPS (*Escherichia coli* 0111:B4; Sigma-Aldrich, St. Louis, MO) was diluted in saline at a dose of 2 mg/50μL and injected into trachea with a microsyringe when mice were under anesthesia; sham-treated mice were given normal saline (NS) alone, described previously [46]. To investigate the role of DNase I on airway inflammation and mucus hypersecretion, mice received aerosolized DNase I (Sigma-Aldrich) 120 U diluted in 5mL NS at 4 and 12 hours after LPS injection through an atomization inhaler. Mice were killed 24 hours after LPS injection.

### Collection of bronchoalveolar lavage fluid (BALF) and lung tissues

Mice were killed by peritoneal injection of pentobarbital sodium (150 mg/kg) 24 hours after LPS injection. The tracheae were cannulated by a 20-gauge catheter and the lungs were lavaged with 0.5mL of ice-cold NS each time for three consecutive washes. A total of 1.2 to 1.4 mL of BALF was recovered and stored at −20°C until analysis. The left lung lobes were fixed with 4% paraformaldehyde (PFA) and used to perform H&E, AB-PAS and immunofluorescence staining. The right lung lobes were stored at −80°C for subsequent RNA and protein extraction.

### Histology, mucus staining and immunofluorescence

The left lung lobes were fixed in 4% PFA for 48 hours and then routinely processed to paraffin blocks and sectioned at approximately 4μm. H&E staining of lung tissue sections was performed to evaluate the inflammatory response. We stained mucus and mucus-containing goblet cells in the bronchial epithelium with an AB-PAS staining kit (Sigma-Aldrich). To detect NETs in lung tissue, paraffin-embedded mouse lung sections were permeabilized with 0.2% Triton X-100 in phosphate-buffered saline (PBS) for 15 minutes and blocked with 2% donkey serum. The sections were then incubated with the primary antibodies—anti-mouse myeloperoxidase (1:100; R&D Systems) and anti-citrullinated histone 3 (1:150; Abcam) overnight at 4°C. Then slides were incubated for 2 hours with secondary antibodies: Alexa Fluor 488 donkey anti-rabbit (1:1000; Abcam) and Alexa Fluor 647 donkey anti-goat (1:1000; Abcam). We used 4′, 6-diamidino-2-phenylindole (DAPI) to detect DNA. Finally, slides were visualized using a confocal microscope.

### Inflammatory cell, cytokine and mucin secretion in BALF

To analyze the differential cell counts in BALF, the BALF was centrifuged (2500 rpm, 4°C, 10 minutes) and the sediment cell pellet in BALF was resuspended in 500 μL of NS and stained using Wright-Giemsa Stain reagent (Sigma-Aldrich) following the manufacturer’s instructions. A minimum of 400 cells was counted and classified as neutrophils, lymphocytes or macrophages according to morphologic criteria. Supernatants in BALF were stored at −80°C for inflammatory cytokine and mucin analysis. ELISA kits were used to detect the levels of mouse IL-6 (BD Biosciences), TNF-α (BD Biosciences), IL-1β (BD Biosciences), MUC5AC (MyBioSource) and MUC5B (MyBioSource) according to the manufacturer’s protocols.

### MPO-DNA ELISA

To quantify NETs in mouse BALF, we created a capture ELISA on the strength of MPO associated with DNA. First, 5 μg/mL of anti-MPO monoclonal antibody (1:500; ABD Serotec, Cat-No. 0400-0002), a key capturing antibody, was used to coat 96-well plates (75 μL per well) overnight at 4°C. Second, non-specific bindings were blocked by 1% bovine serum albumin. BALF (20 μL) and the peroxidase-labeled anti-DNA monoclonal antibody (1:25; Roche) were added to each well. The plate was incubated for 2 hours in a shaking table at 300 rpm and was washed three times. Finally, 100 μL of peroxidase substrate were added. The absorbance at 405 nm wavelength was measured using Fluostar Optima (BMG Labtech) after 30 minutes of incubation at 37°C in the dark.

### Quantitative real-time reverse transcriptase polymerase chain reaction (RT-PCR)

Total RNA was extracted from the right lung tissues using TRIzol reagent (Invitrogen) and cDNA was generated using the Transcriptor First Strand cDNA synthesis kit (Roche) in accordance with the manufacturer’s protocols. Quantitative RT-PCR was performed using the StepOnePlus PCR system and gene expression assays (Applied Biosystems, Foster City, CA, USA). Primer sequences were designed as follows: 5′-AATGCAGCATCATCAACAGCG-3′ (forward) and 5′-AGGCACAGGCGTCATTCACA-3′ (reverse) for mouse MUC5AC; 5′-CTGTACGGGAACCCTAAGGAAA-3′ (forward) and 5′-ACCGCAAGACAGTCGCATTTA-3′ (reverse) for mouse MUC5B; 5′-TGTGTCCGTCGTGGATCTGA-3′ (forward) and 5′-CCTGCTTCACCACCTTCTTGAT-3′ (reverse) for mouse GAPDH. Data were processed using the standard curve method. MUC5AC and MUC5B gene expression was reported as fold change relative to the expression of the NS group.

### Immunoblot analysis

Lung tissues were homogenized by a homogenizer and lysed using RIPA buffer containing a protease inhibitor cocktail (Roche Diagnostics, Indianapolis, IN). The protein concentration of each group was determined using BCA kits. Protein samples (30 μg) were separated by 10% SDS-polyacrylamide gel electrophoresis and subsequently transferred onto PVDF membranes, which were blocked by 5% skim milk for 1 hour at room temperature. After rinsing three times, the blots were incubated overnight at 4°C with the following primary antibodies: anti-TLR4 (1:1,000; Cell Signaling Technology), anti-NF-κB p65 (1:1,000; Cell Signaling Technology), anti-phospho-IκBα (1:1,000; Cell Signaling Technology), anti-IκBα (1:1,000; Santa Cruz), and anti-Cit-H3 (1:1000, Abcam). The membranes were washed three times, incubated with horseradish peroxidase–conjugated secondary antibodies (1:3,000; Sigma-Aldrich), and then visualized by an enhanced chemiluminescence kit (ECL plus). We used anti-GAPDH (1:2,000; Cell Signaling Technology), anti-β-actin (1:10,000; Sigma-Aldrich) and anti-Histone-H3 (1:1500; Sigma) as internal controls. To measure the relative ratio of protein expression, band intensities were quantified by Image-Lab software (Bio-Rad).

### Human NET isolation and quantification

Peripheral blood samples (50 mL) from 10 healthy volunteers were collected with heparinized tubes. After adding diluted blood onto the Lymphocyte Separation Media (sigma-Aldrich), samples were centrifuged at 800 ×g for 30 minutes at 21°C. The interface, which contained lymphocytes and mononuclear cells, between the top two layers was collected carefully for subsequent experiments. Neutrophils and erythrocytes were deposited at the bottom of the tube. Neutrophils were further separated and purified from erythrocytes using 6% Dextran solution and RBC Lysis Buffer. Purified neutrophils were cultured in phenol red free RPMI 1640 medium (Sigma-Aldrich) and stimulated with 500 nM of PMA for 4 hours on a 150 × 25 mm flat tissue culture dish. Cell culture supernatants were discarded carefully, leaving the layer of NETs and neutrophils adhered at the bottom. After washing each dish and centrifuging, cell-free NETs were obtained and quantified using spectrophotometry. NETs (120 ng/μL) were stored at −20°C for subsequent experiments. For specific methods, please refer to our previously published article [47]. This study was approved by the Ethics Committee of Xiangya Hospital, Central South University, and all subjects signed informed consent to participate in this study.

### Cell culture and cytokine analysis

Human monocyte-derived macrophages (HMDMs) were prepared as previously described [48]. CD14-positive monocytes were separated with MACS CD14 micro beads (Miltenyi Biotec) according to the manufacturer’s instructions. Purified monocytes were plated in 96-well plates and differentiated into macrophages by adding 100 ng/mL of recombinant human macrophage colony-stimulating factor (R&D Systems) into RPMI 1640 medium with 10% fetal bovine serum. HMDMs were allowed to attach for at least 3 hours, and then incubated with either 100 ng/mL of LPS or 20 ng/μL of NETs for 24 hours. Supernatant was collected and applied in ELISAs according to the manufacturer’s protocol. ELISA kits of IL-1β, IL-6 and TNF-α were purchased from Invitrogen.

### Statistical analysis

All data were expressed as the mean ± standard deviation (SD) and statistical analyses were performed using GraphPad software. Statistical significance was determined using analysis of variance (ANOVA) followed by a multiple comparison test with Dunnett adjustment. *P* values of <.05 were considered significant.
